# Low agreement between cardiologists diagnosing left ventricular hypertrophy in children with end-stage renal disease

**DOI:** 10.1186/1471-2369-14-170

**Published:** 2013-08-02

**Authors:** Nikki J Schoenmaker, Johanna H van der Lee, Jaap W Groothoff, Gabrielle G van Iperen, Ingrid ME Frohn-Mulder, Ronald B Tanke, Jaap Ottenkamp, Irene M Kuipers

**Affiliations:** 1 Department of Pediatric Nephrology, Emma Children’s Hospital Academic Medical Center, Amsterdam, Netherlands; 2 Department of Pediatric Clinical Epidemiology, Emma Children’s Hospital Academic Medical Center, Amsterdam, Netherlands; 3 Department of Pediatric Cardiology, Wilhelmina Children’s Hospital University Medical Center Utrecht, Utrecht, Netherlands; 4 Department of Pediatric Cardiology, Erasmus Medical Center Sophia, Rotterdam, Netherlands; 5 Department of Pediatric Cardiology, University Medical Center st. Radboud, Nijmegen, Netherlands; 6 Department of Pediatric Cardiology, Emma Children’s Hospital Academic Medical Center Amsterdam, Amsterdam, The Netherlands; 7 Dialysis Department, Academic Medical Center, A01.247 Meibergdreef 9, Amsterdam, 1105 AZ, The Netherlands

**Keywords:** Agreement, Children, End-stage renal disease, Left ventricular hypertrophy, Reproducibility

## Abstract

**Background:**

Monitoring of the appearance of left ventricular hypertrophy (LVH) by echocardiography is currently recommended for in the management of children with End-stage renal disease (ESRD). In order to investigate the validity of this method in ESRD children, we assessed the intra- and inter-observer reproducibility of the diagnosis LVH.

**Methods:**

Echocardiographic measurements in 92 children (0–18 years) with ESRD, made by original analysists, were reassessed offline, twice, by 3 independent observers. Smallest detectable changes (SDC) were calculated for continuous measurements of diastolic interventricular septum (IVSd), Left ventricle posterior wall thickness (LVPWd), Left ventricle end-diastolic diameter (LVEDd), and Left ventricle mass index (LVMI). Cohen’s kappa was calculated to assess the reproducibility of LVH defined in two different ways. LVH_WT_ was defined as Z-value of IVSd and/or LVPWd>2 and LVH_MI_ was defined as LVMI> 103 g/m^2^ for boys and >84 g/m^2^ for girls.

**Results:**

The intra-observer SDCs ranged from 1.6 to 1.7 mm, 2.0 to 2.6 mm and 17.7 to 30.5 g/m^2^ for IVSd, LVPWd and LVMI, respectively. The inter-observer SDCs were 2.6 mm, 2.9 mm and 24.6 g/m^2^ for IVSd, LVPWd and LVMI, respectively. Depending on the observer, the prevalence of LVH_WT_ and LVH_MI_ ranged from 2 to 30% and from 8 to 25%, respectively. Kappas ranged from 0.4 to 1.0 and from 0.1 to 0.5, for intra-and inter- observer reproducibility, respectively.

**Conclusions:**

Changes in diastolic wall thickness of less than 1.6 mm or LVMI less than 17.7 g/m^2^ cannot be distinguished from measurement error in individual children, even when measured by the same observer. This limits the use of echocardiography to detect changes in wall thickness in children with ESRD in routine practice.

## Background

Cardiovascular disease is the leading cause of death in patients with end-stage renal disease (ESRD) [1,2]. In Juvenile ESRD, the mortality associated with cardiac disease is reported to be 500–1000 times higher than in the general age-matched population [3]. Left ventricular hypertrophy (LVH) is an indicator of cardiovascular disease and is independently associated with an increased mortality in adults with ESRD [4]. Echocardiography studies have shown that patients with ESRD have abnormalities of both left ventricular (LV) structure and function [5]. Several factors may be responsible for cardiac disease in ESRD, such as hypertension, anemia, hyperphosphatemia, and a high parathyroid hormone (PTH) [6]. In adult-onset ESRD, therapeutic interventions, such as an increase of the dialysis frequency and a stricter control of hypertension, hyperphosphatemia and anemia have been investigated to reduce cardiac mortality in patients with LVH [7]. Recently, Mitsnefes et al. emphasized the importance of frequent dialysis in children [8]. Small single-center studies have shown clinical improvements in LVH and function when children receive dialysis more frequently than the traditional, thrice-weekly schedule [9,10]. Timely detection of cardiovascular disease in children with ESRD would therefore give an opportunity for targeted intervention in the high-risk patients, thereby preventing cardiovascular morbidity and mortality in early adulthood.

LVH, diagnosed with echocardiography, is considered to be a reliable surrogate outcome marker for cardiac disease in ESRD. Only recently, Chavers et al. have proposed the performance of periodic routine echocardiography in all children with ESRD, to detect cardiac disease at an early stage [11]. In the Netherlands, Belgium and Germany, the most widely used definition of LVH in children is based on the M-mode echocardiographic measurement of the interventricular septum thickness (IVSd) and left ventricular posterior wall in diastole (LVPWd). In the Netherlands, there are normal values for Dutch healthy children according to weight [12]. In many clinical trials and epidemiological studies LVH was defined based on the LV Mass (LVM) [13,14]. The interpretation of the LVM in children is challenging because such values need to be appropriately indexed for body size. Various different methods of indexing LVM to body size have been reported, including adjustment for body surface area (BSA) [15] and height^2.7^[16,17]. The definition of LVH is a matter of ongoing controversy [17,18], especially in possibly growth-retarded children [19,20].

In a project on quality assessment in children with end-stage renal disease, the RICH-Q project (Renal Insufficiency Therapy in Children: Quality Assessment and Improvement) [21], in which all Dutch centers for pediatric renal replacement therapy participate, we observed an unexpectedly large variation in the prevalence of LVH, using one definition, among all the Dutch centers. This raised questions about the reliability of the measurements. We therefore decided to investigate the intra- and inter-observer reproducibility of the measurements of left ventricular wall thicknesses during echocardiography and of the diagnosis LVH.

## Methods

### Study participants

We included all children (0–18 years) treated with renal replacement therapy (RRT) between August 1^st^ 2007 and January 1^st^ 2011 in the Netherlands in the study. Written informed consent from the parents and/or the participants and approval from the ethical boards of all participating hospitals (Academic Medical Center, Amsterdam, Erasmus Medical Center Rotterdam, University Medical Center St Radboud Nijmegen) were obtained. For patients whose parents have provided informed consent, data on the routinely performed pediatric echocardiography are registered centrally in the RICH-Q project [21].

### Data collection

We reviewed one echocardiogram per subject. The recording and analysis of the echocardiograms had been performed by pediatric cardiologists (“original analysts”) in all 4 Dutch centers using either Vivid 7 (GE Medical Systems, Wauwatosa, WI) or Philips Sonos 5500, (Medical Systems, Andouver) ultrasound systems. Two-dimensionally guided M-mode echocardiography was performed from a parasternal long- axis view. Interventricular septum thickness in diastole (IVSd) in mm, left ventricular posterior wall thickness in diastole (LVPWd) in mm and left ventricular end-diastolic diameter (LVEDd) in mm were measured at end-diastole at the level just below the mitral valve leaflets. A simultaneous electrocardiogram (ECG) was used for the timing of the measurements in the cardiac cycle. The diastolic wall thickness was measured at the onset of the QRS wave of the ECG [22]. Digital images were stored and analyzed offline in the Emma Children's Hospital, Amsterdam, using the same device (EchoPac 108.1.5 General Electric Medical Systems, by three independent observers. Observers 1 and 2 were pediatric cardiologists, each with more than 12 years of experience in interpreting pediatric echocardiograms. Observer 3 was a research physician specifically trained in echocardiography. Each variable, i.e. IVSd, LVPWd and LVEDd, was measured three times and the mean was calculated.

LV mass was calculated using the following equation: LV mass (grams) = 0.8 (1.04 ([LVEDd + IVSd + LVPWd]^3^ – [LVEDd]^3^)) + 0.6 g [23]. To account for body size, the LV mass index (LVMI) (g/m^2^) was calculated by dividing LV mass by body surface area (BSA). Two definitions were used for diagnosing LVH, one is based on the wall thicknesses of IVSd and LVPWd (LVH_WT_) and the other on LVMI (LVH _MI_). LVH_WT_ was defined as a Z-score of either IVSd or LVPWd > + 2 based on a set of normal values of healthy Dutch children, corrected for weight, from Overbeek et al. [12]. LVH_MI_ was defined as LVMI > 103 g/m^2^ for boys and > 84 g/m^2^ for girls [16]. Observers 1, 2 and 3 reassessed all echocardiograms in a randomly different order after a period of at least two weeks to preclude recollection of the values. This was the first part of the study.

Usually the echocardiographic measurements are made by a cardiologist who stores 3 images, each representing 3 heart cycles. Each image is accompanied by the corresponding ECG. The cardiologist chooses one of the 3 onsets of the QRS wave of the ECG in 1 of the 3 images for measurement of the diastolic wall thickness which he considers appropriate for measurement. This procedure is based on the presumption that the variation between the M-mode images is of no influence on the measurement of the wall thickness. If this presumption would not be true, exclusion of this source of variation might improve the reproducibility of the measurement. Therefore we designed the second part of the study, in which observers 1 and 2 assessed one specific pre-selected image from each of twenty echocardiograms twice with a period of at least two weeks in between. The 20 echocardiograms were selected based on the range of Z-scores of IVSd, LVPWd and LVEDd to represent the entire range of the wall thickness in the patient population. From each echocardiogram, observer 2 selected one exact point of one heartbeat cycle in 1 image. Both observers measured IVSd, LVPWd and LVEDd at exactly the same point in that specific image three times and the mean was calculated.

### Statistical analysis

Intra- and inter-observer reproducibility of the continuous measures IVSd, LVEDd, LVPWd and LVMI was assessed by the Bland Altman method. The limits of agreement (LoA) are calculated as the mean difference in scores of repeated measurements (mean) ± 2 × standard deviation of these differences (SD_diff_) [24]. The variability of the measurement is also reported as Smallest Detectable Change (SDC), calculated as 1.96 × √2 × standard error of measurement (SEM). SEM is the square root of the error variance calculated by analysis of variance (ANOVA). SDC reflects the smallest within-person change in score that, with *P* < 0.05, can be interpreted as a “real” change, above measurement error, in one individual (SDC_ind_) [24].

Cohen’s kappa was calculated to assess intra- and inter-observer agreement for dichotomous variables, i.e. presence or absence of LVH. The interpretation of kappa is arbitrary. A value of 0.70 is generally recommended as a minimum standard for reliability [25].

## Results

Ninety-two children with ESRD (0–18 years old) were included. Fifty one (55%) were boys; the median (range) age, weight and BSA were 13.1 (0.7- 19.1) years, 37.8 (4.3- 96.0) kg and 1.2 (0.4- 2.2) m^2^, respectively (Table 1). The median (range) duration of RRT was 1.8 (0–6.8) years. At time of the echocardiography, 26 children (28%) were treated with hemodialysis, 24 (26%) with peritoneal dialysis and 42 (46%) were transplanted.

**Table 1:** Demographics of children with End-stage renal disease at time of the Echocardiography

	**Children with ESRD**
	**(n=92)**
Age (years)	13.1 (0.7-19.1)
Male*	51 (55%)
Weight (kilograms)	37.8 (4.3-96.0)
BSA (m^2^)	1.2 (0.4-2.2)
Height (cm)	138 (61–184)
Duration of RRT (years)	1.8 (0.0-6.8)
Modality at time of Echocardiography*	
-Hemodialysis	26 (28%)
-Peritoneal dialysis	24 (26%)
-Transplantation	42 (46%)

Data are presented as median (range), *Data are presented as n (%), *BSA* Body surface area.

All 4 observers (the original analyst, observers 1, 2 and 3) agreed in 2 patients diagnosing LVH and in 46 patients by diagnosing non LVH_WT_. In Table 2 we compared the demographics for these 48 children the observers agreed on vs. the other 44 children the observers could not agree on the diagnosis LVH_WT_ or no-LVH_WT_ (Table 2). There were no significant differences found between the 2 groups

**Table 2:** **Demographics of children who were diagnosed with LVH**_**WT **_**or no LVH**_**WT **_**by all 4 observers vs. all children were there was no agreement (n=44)**

	**All agree on LVH**_**WT **_**or no LVH**_**WT**_	**No agreement on LVH**_**WT **_**or no LVH**_**WT**_	**P value**
	**n=48**	**n=44**	
Age (years)	12.6 (1.4-18.3)	14.0 (0.7-19.1)	0.23^a^
Male*	23 (49%)	28 (62%)	0.14^b^
Weight (kilograms)	33.0 (4.3-96.0)	41 (7.0-88.5)	0.44^a^
BSA (m^2^)	1.2 (0.4-2.2)	1.3 (0.4-2.1)	0.43^a^
Height (cm)	145 (85–181)	153 (69–184)	0.52^a^
Duration of RRT (years)	1.6 (0–6.8)	1.7 (0–5.9)	0.70^a^
Modality at time of Echocardiography*			0.54 ^b^
-Hemodialysis	14 (29%)	12 (27%)	
-Peritoneal dialysis	11 (23%)	13 (30%)	
-Transplantation	23 (48%)	19 (43%)	

Data are presented in median (range), * Data are presented as n (%), *RRT* renal replacement therapy, ^a^ mann-Withney U, ^b^ chi square test.

The results of the intra- and inter-observer reproducibility are shown in Table 3.

**Table 3:** Intra- and inter-observer reproducibility of echocardiographic measurements

**Intra-observer**		**Observer 1**	**Observer 2**	**Observer 3**
IVSd (mm)	Median (range)	7.0 (3.8-11.0)	6.1 (3.8-10.5)	6.9 (3.7-12.8)
	Mean diff. [95% CI]	0.2 [0.0-0.4]	0.1 [0.0-0.3]	0.1 [-0.1-0.3]
	LoA	-1.6-1.9	-1.4-1.7	-1.6-1.8
	SDC	1.7	1.6	1.7
LVPWd (mm)	Median (range)	7.0 (4.2-12.0)	5.9 (3.4-10.7)	7.9 (4.1-14.2)
	Mean diff. [95% CI]	0.4 [-0.1-0.7]	0.3 [-0.3-0.8]	0.1 [-0.1-0.4]
	LoA	-2.1 - 2.9	-1.1-1.7	-2.0 -2.1
	SDC	2.5	2.0	2.6
LVEDd (mm)	Median (range)	41 (21–59)	44 (23–63)	41 (20–57)
	Mean diff. [95% CI]	0.9 [0.2-3.2]	0.2 [-0.8-0.5]	0.2 [-0.3-0.6]
	LoA	-6.1 - 7.9	-2.7- 3.1	-4.3 - 4.4
	SDC	6.9	2.8	4.4
LVMI	Median (range)	76 (34–219)	70 (39–181)	80 (31–255)
(g/m^2^)	Mean diff. [95% CI]	1.1 [-2.1-4.3]	0.7 [-1.2-2.5]	0.7 [-1.5-2.9]
	LoA	- 30.1 – 32.3	-17.4 – 18.8	-20.5 – 21.9
	SDC	30.5	17.7	20.7
LVH_WT_	Kappa [95% CI]	0.4 [0.2-0.7]	1.0 [1.0-1.0]	0.4 [0.2-0.6]
LVH_MI_	Kappa [95% CI]	0.6 [0.4-0.8]	0.6 [0.3-0.9]	0.5 [0.3-0.7]
Inter-observer		Observer 1 vs 2	Observer 1 vs 3	Observer 2 vs 3
IVSd (mm)	Mean diff. [95% CI]	1.0 [0.7-1.2]	0.1 [0.0-0.3]	1.1 [0.9-1.3]
	LoA	1.3 – 3.3	-1.6 - 1.8	-1.1 – 3.3
	SDC (3 observers)	2.6		
LVPWd (mm)	Mean diff. [95% CI]	1.0 [0.8-1.2]	0.6 [0.4-0.8]	1.6 [1.3-1.8]
	LoA	-1.0 – 3.0	-1.4 – 2.4	-0.8 – 3.9
	SDC (3 observers)	2.9		
LVEDd (mm)	Mean diff. [95% CI]	2.4 [1.9-2.9]	0.3 [-0.4-1.0]	2.7 [2.0-3.4]
	LoA	-2.1 – 6.9	- 6.4 - 7.2	- 3.8 – 9.3
	SDC (3 observers)	5.9		
LVMI (g/ m^2^ )	Mean diff. [95% CI]	6.5 [3.9-9.2]	4.0 [1.6-6.4]	10.6 [7.8-13.3]
	LoA	-6.2 – 31.9	-19.1 – 27.2	- 15.7 – 37.2
	SDC (3 observers)	24.6		
LVH_WT_	Kappa [95% CI]	0.1 [-0.2-0.5]	0.4 [0.2-0.6]	0.1 [-0.2-0.4]
LVH_MI_	Kappa [95% CI]	0.3 [0.1-0.5]	0.5 [0.3-0.7]	0.4 [0.2-0.7]

Part 1 (n=92).

*IVSd* diastolic interventricular septum, *LVEDd* left ventricular end-diastolic diameter, *LVPWd* diastolic left ventricular posterior wall thickness, *LVMI* left ventricular mass index, *Mean diff*, mean difference, *CI* confidence interval, *LoA* limits of agreement, *SDC* smallest detectable change, LVH_WT_ was defined as a Z-score of IVSd and\or LVPWd > + 2 (according to normal values of healthy Dutch children), LVH_MI_ was defined as LV mass index (g/m^2^) > 103 g/m^2^ for boys and > 84 g/m^2^ for girls.

The IVSd and LVPWd measurements of observer 2 were consistently smaller than those of observers 1 and 3. The intra-observer SDCs ranged from 1.6 to 1.7 mm, from 2.0 to 2.6 mm, from 2.8 to 6.9 mm and from 17.7 to 30.5 g/m^2^ for IVSd, LVPWd, LVEDd and LVMI, respectively. The intra-observer kappas for the 3 observers for LVH_WT_ and LVH_MI_ ranged from 0.4 to 1.0 and from 0.5 to 0.6, respectively.

The inter-observer SDCs were 2.6 mm, 2.9 mm, 5.9 mm and 24.6 g/m^2^ for IVSd, LVPWd, LVEDd and LVMI, respectively. The inter-observer kappas for LVM_WT_ and LVH_MI_ ranged from 0.1 to 0.4 and from 0.3 to 0.5, respectively.

### Selected image of 20 selected patients

From the 20 children whose echocardiograms were selected for the second part of the study, 10 (50%) had been diagnosed with LVH_WT_ by the original observer. The results of the intra- and inter-observer reproducibility study are shown in Table 4.

**Table 4:** Intra- and inter-observer reproducibility based on identical images

**Intra-observer**		**Observer 1**	**Observer 2**
IVSd (mm)	Median (range)	7.5 (4.2-11.9)	7.4 (3.7-11.6)
	Mean diff. (95% CI)	0.2 (-0.2- 0.6)	0.4 (0.0-0.8)
	LoA	-1.4-1.8	-1.2-2.1
	SDC	1.6	1.8
LVPWd (mm)	Median (range)	6.9 (3.9-12.3)	7.1 (4.0-11.3)
	Mean diff. (95% CI)	0.4(-0.3-1.1)	0.5 (0.1-0.9)
	LoA	-2.5-3.4	-1.3-2.3
	SDC	3.3	2.0
LVEDd (mm)	Median (range)	43.2 (26.3-57.2)	43.9 (26.3-57.2)
	Mean diff. (95% CI)	0.4 (-0.3-1.0)	0.6 (0.1-1.1)
	LoA	-2.6-3.3	-1.6-4.9
	SDC	2.9	2.2
LVMI	Median (range)	79 (45–226)	82 (47–221)
(g/m^2^)	Mean diff. (95% CI)	4.0 (-1.4-9.4)	4.7 (0.8-8.7)
	LoA	-19.2-27.2	-12.2-21.7
	SDC	22.8	16.6
Inter-observer		Observer 1 vs 2	
IVSd (mm)	Mean diff (95% CI)	0.4 (0.0-0.7)	
	LoA	-1.1-1.9	
	SDC	1.6	
LVPWd (mm)	Mean diff. (95% CI)	0.3 (-0.1-0.8)	
	LoA	-1.57-2.25	
	SDC	1.9	
LVEDd (mm)	Mean diff. (95% CI)	1.5 (0.7-2.3)	
	LoA	-2.0-4.9	
	SDC	3.4	
LVMI	Mean diff. (95% CI)	0.1 (-5.3-5.6)	
(g/m^2^)	LoA	-23.3-23.5	
	SDC	22.9	

Part 2 (n=20).

*IVSd* diastolic interventricular septum, *LVEDd* left ventricular end-diastolic diameter, *LVPWd* diastolic left ventricular posterior wall thickness, *LVMI* Left ventricular mass index, *Mean diff* mean difference, *CI* confidence interval, *LoA* limits of agreement, *SDC* smallest detectable change.

None of the reproducibility results were superior to those of the first part of the study, in which each observer chose the images to be measured.

The results of the assessment of diagnosing LVH by the original analyst, observers 1, 2 and 3 of all 92 echocardiograms are shown in Table 5. The prevalence of LVH_WT_ and LVH_MI_ ranged from 2 to 30% and from 8 to 25% of the 92 patients, respectively. All 4 observers (the original analyst, observers 1, 2 and 3) agreed in 46 patients and 56 patients by diagnosing non LVH_WT_ and non LVH_MI_, respectively. All 4 observers agreed in 2 patients and in 6 patients by diagnosing LVH_WT_ and LVH_MI,_ respectively. Use of the LVH_WT_ definition resulted in a higher prevalence for two observers and a lower prevalence for the other two observers and vice versa, indicating that there is not a simple relation between the two definitions.

**Table 5:** Prevalence of LVH according to different definitions and different observers

**Observer**	**LVH**_**WT**_**^ (%)**	**LVH**_**MI**_^**# **^**(%)**	**Kappa between LVH**_**WT **_**and LVH**_**MI**_
	**n=92**	**n=92**	
Original analyst*	28 (30%)	22 (24%)	0.62
1	20 (22%)	23 (25%)	0.46
2	2 (2%)	7 (8%)	0.42
3	27 (29%)	22 (24%)	0.42

*The treating cardiologist who made the echocardiogram, *LVH* left ventricular hypertrophy, ^LVH_WT_ was defined as a Z-score of IVSd and/or LVPWd > + 2 (according to normal values of healthy Dutch children), ^#^LVH_MI_ was defined as LV mass index >103 g/m^2^ for boys and >84 g/m^2^ for girls.

## Discussion

We found a low reproducibility of the measurement of ventricular wall thickness, and as a result low agreement within and between observers in diagnosing LVH using conventional echocardiography in children with ESRD. The inaccurateness of the wall thickness measurements affects LVH assessment for all different definitions of LVH. Our data demonstrate that in individual children, changes in diastolic wall thickness or LVMI as a result of ESRD which are expected over a period of a year cannot be distinguished from measurement error, even when measured by the same observer.

With more than 30 years of clinical use, echocardiography has become one of the most important non-invasive imaging methods in the evaluation of cardiac morphology and dynamics. It is generally considered a valuable method for the detection of LVH, due to its wide availability, non-invasiveness, and relatively low cost. However, in children the interpretation of echocardiographic data is hampered by various problems.

First, according to our study, the variability of outcomes even within one experienced observer is of such magnitude that expected changes in wall thickness can not reliably be monitored reliably. The intra-and inter-observer SDCs of the echocardiographic measurement of the IVSd and the LVPWd ranged from 1.6 to 2.6 mm and 2.0 to 2.9 mm, respectively. In an individual child only changes in septum or wall thickness larger than the SDC can be considered as “real change”. Since in children the normal values of IVSd and LVPWd are small, ranging from 3 to 10 mm and from 3 to 13 mm respectively, changes over time in an individual child need to be as large as 16 to 100% of the normal value before they can be considered as “real change”, i.e., not due to measurement variability. The intra-and inter-observer SDC of the LVMI ranged from 17.7 to 30.5 g/m^2^. Similarly, since in children the normal values of LVMI are 40 to 80 g/m2, changes over time in an individual child need to be as large as 30-60% of the normal value before they can be considered as “real change”.

Secondly, there is little consensus about the definition of LVH in children. In adults LVH is usually defined as LVMI > 51 (g/m^2.7^), which is associated with increased cardiovascular morbidity and mortality [26]. In children, LVH is based on the normal distribution of LVMI or wall thickness in healthy children, because cardiovascular outcome studies in children are lacking due to the low incidence of cardiovascular events. Some physicians define LVH as LVM according to Devereux corrected for body size above a cutoff value (> 51 g/m^2.7^ or 38.6 g/m^2.7^) or above the P95 of healthy children for age. Yet, different indexations with respect to body size are used. All these different indexes for LVM lead to important differences in LVH prevalence, varying from 18% to 55% in children with chronic kidney disease [20] and from 27% to 52% in children on peritoneal dialysis [19]. In 2009 Khoury et al. reported normal values for LVM indexed for height in children between 0 and 18 years of age [18]. As LVM indexed for weight or BSA had been found not to be accurate in children with obesity [27], it is conceivable that Khoury’s normal values are not applicable in growth-retarded (ESRD) children either. Borzych et al. showed that when using the Khoury charts, LVH prevalence was significantly higher in growth-retarded children on peritoneal dialysis than in children on peritoneal dialysis of normal height [19]. Another approach to the definition of LVH is using z-scores of only the septal and/or posterior wall thickness as measure for LVH. In this definition, the effect of changes in left ventricular end-diastolic volume is neglected. Since ventricular dilatation leads to wall thinning, LVH may be missed in dilated cardiomyopathy by this method. In the present study we used LVM indexed for BSA because in our opinion height and weight allow a better estimation of lean body mass, and therefore heart size, than height^2.7^ in children with possible growth retardation [17].

Our results are in line with other studies. In two studies in healthy children, intra-observer variance was found to be smaller than inter-observer variance, as is usually the case [28,29]. In the study by Schieken et al. [28] the intra-observer SDC was 1.7 mm for both IVSd and LVPWd. An explanation for this relatively high reproducibility might be that the data from the Schieken et al.’s study were derived from a selection of 20 out of 28 echocardiograms, which satisfied the criteria for technical acceptability. Inter-observer SDCs were comparable with our findings. In the study by Day et al. [29] the intra-observer mean differences were equal to those found in our study, but the inter-observer mean differences were considerably larger. Still, the authors concluded that “the echocardiographic measurements taken from healthy children in a longitudinal study can be made accurately with acceptable reproducibility”.

The reproducibility of the LV mass is highly dependent on the reproducibility of IVSd and LVPWd. In M-mode measurements, differences of IVSd and LVPWd of approximately 5% may be translated into differences in LV mass between 8% and 15%, which may represent about 50 g in adults [30]. Thus, measurement inaccuracies in individual adult patients limit the use of the Devereux formula to calculate LV mass [30]. Test-retest studies in adults indeed found differences of up to 30 g between tests [31,32]. Therefore the authors of ‘the reliability of M-Mode echocardiography studies’ (the RES trial) concluded that the probability of a true change in LV mass over time is maximized for a single-reader difference greater than 18% of the initial value.

Theoretically there are several sources of variability that can influence echocardiographic measurements. In our study the following sources of variation were excluded by the design of offline assessment of the non-moving stored images: placing of the echocardiogram probe, the way the images are captured and obviously within-patient variability like day-to-day variability in fluid status (e.g. before or after dialysis) and blood pressure. To minimize other sources of variation, such as timing in the cardiac cycle, all measurements were made according to the Guidelines and Standards for Performance of a Pediatric Echocardiogram of the American Society of Echocardiography^7^. In these guidelines, the exact choice of the images and heart cycles within one assessment on which the calculations are based is not defined. We therefore adjusted the protocol in part two of the study to exclude any potential variability due to the choice of the image and heart cycle. This did not lead to improvement of the reproducibility results, indicating that the variability is inherent in the measurement procedure, and not due to the choice of the particular image.

To improve reproducibility the standard method is to measure multiple heart cycles and calculate the mean value, as was done in our study. In addition, some authors even advise interpretation by more than one cardiologist [33]. Others, however, advise that the same cardiologist reads all the echocardiograms for an individual patient to reduce the variability of the longitudinal measurements [34]. This is unfortunately not supported by the low intra-observer reproducibility that we found. In 2010 Lopez et al. developed recommendations for quantifications during the performance of a pediatric echocardiogram [35]. However, these recommendations are based on 2D or 3D short axis imaging, while in Europe M-mode echocardiography is still the most used technique. To date, it has to be established if 2D or 3D echocardiography is indeed better reproducible than M-mode echocardiography. Magnetic resonance imaging (MRI) of the heart could be valuable for diagnosing LVH, as it is more accurate and reproducible than echocardiography [36]. It can be used to precisely estimate a patient's left ventricular mass and assess other structural cardiac abnormalities. Contrary to echocardiography, MRI has several disadvantages that preclude its use in daily clinical practice. It is expensive, time consuming, not easily accessible and obviously not bed side. Furthermore, in young children sedation is often necessary.

A limitation of this study is that the original echocardiograms were assessed retrospectively without the original observers knowing this would be a reproducibility study. The observers 1, 2 and 3 were aware of the fact this was a reproducibility study. This could be an explanation of the differences between the original observer and the other observers. Another limitation of this retrospective design is that not all original echocardiograms were performed at equal time intervals after a hemodialysis session for the 26 children on hemodialysis. Although the reproducibility measurements were assessed by the exact same offline images, the wall thickness could be affected by fluid overload. Finally, although observers 1, 2 and 3 used the same device to evaluate the images, we cannot exclude potential variation due to the use of the Vivid 7 or Philips Sonos 5500 for the original echocardiography.

Furthermore, Cohen’s kappa gives a quantitative assessment of how well two raters agree corrected for chance agreement. The interpretation of kappa is arbitrary. Several difficulties have been described with the interpretation of kappa, however, one of which is related to the prevalence of the condition [37,38]. If only the most severe cases are diagnosed as LVH as was done by observer 2, the intra-observer kappa is inflated.

## Conclusions

Our study has important clear implications. The need for cardiovascular monitoring in children with ESRD is beyond discussion. Timely detection of left ventricular abnormalities may decrease the risk for early cardiac death by therapeutic adjustments such as more frequent dialysis, conversion from peritoneal dialysis to (frequent) hemodialysis, dietary measures or adjustment of medication. In this respect, routinely yearly echocardiography in children with ESRD has been promoted. Yet we believe that LVH assessment by conventional echocardiography in an individual child with ESRD may too easily result in either underreporting of LVH or in an spurious diagnosis of LVH. This may either lead to a potentially preventable deterioration of cardiac function or to unnecessary interventions with potential burdens for the patient. We therefore believe that new, more sensitive tools (e.g. MRI, 3D echocardiography, Tissue Doppler imaging and Speckle Tracking Echocardiography) need to be explored as reliable tools for longitudinal cardiac follow-up in these children.

## Abbreviations

BSA: Body surface area; CI: Confidence interval; ECG: Electrocardiogram; ESRD: End-stage renal disease; IVSd: Diastolic interventricular septum; LoA: Limits of agreement; LVEDd: Left ventricular end-diastolic diameter; LVH: Left ventricular hypertrophy; LVHMI: was defined as LV mass index (g/m^2^) > 103 g/m^2^ for boys and > 84 g/m^2^ for girls; LVHWT: was defined as a Z-score of IVSd and\or LVPWd > + 2 (according to normal values of healthy Dutch children); LVM: Left ventricular mass; LVMI: Left ventricular mass index; LVPWd: Diastolic left ventricular posterior wall thickness; Mean diff: Mean difference; PTH: Parathyroid hormone; RRT: Renal replacement therapy; SDC: Smallest detectable change.

## Competing interest

The authors declare that they have no competing interests.

## Authors’ contribution

Each author has participated sufficiently in the work to take public responsibility for the content. Conception of the manuscript, analysis, and interpretation of data were performed by NS, IK, JvdL and JG. The other authors represent the participating centres and made the original echocardiographic images. NS, IK and JO carried out the reproducibility study. All authors read and approved the final manuscript.

## Pre-publication history

The pre-publication history for this paper can be accessed here:

http://www.biomedcentral.com/1471-2369/14/170/prepub
